# Association between inflammation factors and *Mycoplasma pneumoniae* in children: Protocol for a systematic review

**DOI:** 10.1097/MD.0000000000015118

**Published:** 2019-04-12

**Authors:** Jin-e He, Hui Qu, Chun-Yan Gao

**Affiliations:** Third Unit of Pediatric Department, Yanan University Affiliated Hospital, Yan’an, China.

**Keywords:** cytokines, factors, interleukin, *Mycoplasma pneumoniae*

## Abstract

**Background:**

Several clinical studies have reported that inflammation factors (IF) are associated with *Mycoplasma pneumoniae* in children. However, no study systematically investigated the association between IF and *M pneumoniae* in pediatric population. Thus, this study will explore the association between IF and pediatric *M pneumoniae* systematically.

**Methods:**

This study will search following databases of PUBMED, PsycINFO, Scopus, Cochrane Library, EMBASE, Web of Science, and Chinese Biomedical Literature Database from inception to the February 28, 2019 without any language limitations. We will cover clinical studies of *M pneumoniae* that report associations between IF and *M pneumoniae*. In addition, reference lists of relevant studies will also be identified to avoid missing any eligible studies. Two investigators will independently screen and select studies, and will assess the methodological quality for each study, which is evaluated by using Newcastle Ottawa Scale. Any disagreements will be settled down through discussion with a third investigator until consensus is reached.

**Results:**

This study will explore the associations between IF and *M pneumoniae* by assessing the changes of IF, such as interleukin (IL)-4, IL-5, IL-6, IL-10, IL-13, and IL-17 at different stages of *M pneumoniae*.

**Conclusion:**

The findings of this study may provide most recent evidence for the associations between IF and *M pneumoniae* in pediatric populations.

**Ethics and dissemination:**

Ethical approval is not needed in this study, because no individual patient data will be utilized in this study. The findings of this study are expected to be published at peer-reviewed journal or will be presented at professional conference.

**PROSPERO registration number:**

PROSPERO CRD42019125359.

## 1 Introduction

*Mycoplasma pneumoniae* is a common respiratory pathogen that is responsible for the community-acquired pneumonia (CAP), especially in children.^[1–3]^ Furthermore, it also triggers the exacerbation of asthmatic symptoms and wheezes in children.^[4–9]^ It has been reported that *M pneumoniae* accounts for 7% to 40% of all CAP in children 3 to 15 years of age.^[10]^ Fortunately, it has a lower incidence in children under 3 years old.^[10]^ Other respiratory conditions are also reported to have association with *M pneumoniae*. These conditions often include tracheobronchitis, bronchopneumonia, pharyngitis, sinusitis, croup, and bronchiolitis.^[11]^

Although the clinical significance of *M pneumoniae* infection is becoming evident, its pathophysiological mechanisms of serum inflammation factors (IF) in children still have not been fully understood. Several cytokines are reported to have associated with *M pneumoniae*.^[12–18]^ These cytokines consist of interleukin (IL)-4, IL-5, IL-6, IL-10, IL-13, and IL-17.^[12–18]^ However, up to the present, no systematic review has been addressed to explore the associations between IF and *M pneumoniae* in pediatric population. Therefore, this study will firstly explore the associations between IF and *M pneumoniae* in pediatric patients.

## 2 Methods

### 2.1 Study registration

This study has been registered on PROSPERO (CRD42019125359) and has reported according to the guidelines of Preferred Reporting Items for Systematic Reviews and Meta-Analysis Protocol (PRISMA-P) statement.^[19]^

### 2.2 Eligibility criteria for study selection

#### 2.2.1 Types of studies

All randomized controlled trials (RCTs), observational studies or case-control studies will all be considered for inclusion in this study. However, non-clinical studies, case reports, case series will not be considered.

#### 2.2.2 Types of participants

All pediatric patients with age ***<***18 years old, and are clinically diagnosed with *M pneumoniae*, and have checked by IF, such as IL-4, IL-5, IL-6, IL-10, IL-13, and IL-17. Participants will be excluded if they are accompanied with other chronic respiratory diseases or disorders, such as cystic fibrosis, bronchiectasis, bronchopulmonary dysplasia, or immunodeficiency.

#### 2.2.3 Types of exposures

Exposure includes IF following *M pneumoniae* will be considered as experimental exposures. Comparators are a group of participants without *M pneumoniae*.

#### 2.2.4 Types of outcomes

The outcome measurements include any IF, such as IL-4, IL-5, IL-6, IL-10, IL-13, and IL-17.

### 2.3 Literature sources and search methods

#### 2.3.1 Search strategy

We will comprehensively search the literature sources of PUBMED, PsycINFO, Scopus, Cochrane Library, EMBASE, Web of Science, and Chinese Biomedical Literature Database from inception to February 28, 2019 without any language restrictions. Additionally, reference lists of relevant studies will also be searched to avoid missing any potential studies. The detailed search strategy for Cochrane Library is presented in Table 1. Similar detailed search strategies will also apply to any other electronic databases.

**Table 1:**
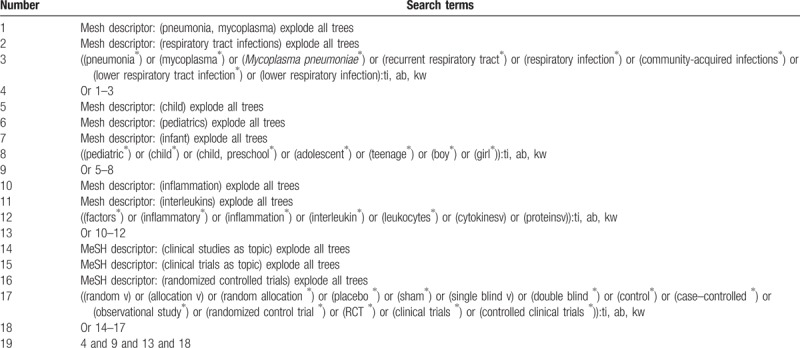
Search strategy applied in Cochrane Library database.

#### 2.3.2 Study selection

Two investigators will independently select the studies on the basis of the predefined eligibility criteria. The study selection will consist of 2 stages. First, all titles and abstracts will be scanned by 2 investigators. Second, both investigators will obtain full-text literature to further check those meet the inclusion criteria. The whole process of study selection is abided to the guidelines of PRISMA-P, and reasons for exclusions and inclusions of all articles will be shown in PRISMA flowchart. Any discrepancies will be resolved by consulting a third investigator through discussion.

#### 2.3.3 Data extraction

All required data will be double extracted by 2 independent investigators using a pre-designed standardized data extraction form. Any disagreements regarding the data extraction will be solved by a third investigator through discussion. Data in detail will be extracted from each study as follows: title, first author name, year of publication, journal, country, study design, patient selection, age, sample size, types of exposures, outcome variables, and any other important information.

#### 2.3.4 Dealing with essential missing information

Missing information or data will be inquired by contacting primary authors. If we can not get those data, we will just analyze the available data and will discuss its impacts as a limitation.

### 2.4 Methodological quality assessment

Methodological quality of each study will be evaluated by using Newcastle–Ottawa Scale checklist.^[20]^ This tool ranges from 0 (lowest quality) to 9 (best quality). Two independent investigators will assess the methodological quality for each study. Any disagreements regarding the methodological quality between 2 investigators will be resolved by consulting a third investigator. Summary risk of bias table will be built.

### 2.5 Statistical analysis

STATA 12.0 software will be used for statistical analysis in this study. If there are sufficient eligible studies, the data will be pooled, and meta-analysis will be conducted. Mean difference with 95% confidence intervals (CIs) will be used to summarize the continuous data. Risk ratio and 95% CIs will be utilized to express the dichotomous data. Heterogeneity across the included studies will be assessed by using *I*^2^ test. The acceptable heterogeneity will be considered if *I*^2^ ≤50%, then data will be pooled by using a fixed-effect model, and meta-analysis will be carried out. The substantial heterogeneity will be regarded if *I*^2^ >50%, and data will be pooled by using a random-effect model. Meanwhile, subgroup analysis will be performed. If substantial heterogeneity is still identified after subgroup analysis, data will not be pooled, and meta-analysis will not be conducted. However, we will still report the results as native summary.

### 2.6 Additional analysis

#### 2.6.1 Subgroup analysis

Subgroup analysis will be performed based on different characteristics, outcome values, and study quality.

#### 2.6.2 Sensitivity analysis

Sensitivity analysis will be operated to check the robustness and stability of pooled outcome results data by removing low-quality studies.

#### 2.6.3 Reporting bias

Funnel plots and Egger regression test will be utilized to check the reporting bias if sufficient studies are included.^[21]^

## 3 Discussion

Several previous clinical studies have reported that IF has associations with *M pneumoniae* in children.^[12–18]^ However, no systematic review and meta-analysis have explored the associations between IF and *M pneumoniae* in pediatric patients. Thus, in this study, we will systematically investigated the associations between IF and *M pneumoniae* in children by searching comprehensive literature databases. The results of the present study will summarize the latest evidence on the associations between IF and *M pneumoniae* in pediatric patients. The findings may also provide helpful evidence for both patients and clinicians.

## Author contributions

**Conceptualization:** Jin-e He, Chun-Yan Gao.

**Data curation:** Jin-e He, Hui Qu, Chun-Yan Gao.

**Formal analysis:** Jin-e He, Hui Qu.

**Funding acquisition:** Jin-e He.

**Investigation:** Chun-Yan Gao.

**Methodology:** Jin-e He.

**Project administration:** Chun-Yan Gao.

**Resources:** Jin-e He, Hui Qu.

**Software:** Jin-e He, Hui Qu.

**Supervision:** Chun-Yan Gao.

**Validation:** Hui Qu, Chun-Yan Gao.

**Visualization:** Jin-e He, Hui Qu, Chun-Yan Gao.

**Writing – Original Draft:** Jin-e He, Hui Qu, Chun-Yan Gao.

**Writing – Review & Editing:** Jin-e He, Hui Qu, Chun-Yan Gao.

## References

[1] Kumar S *Mycoplasma pneumoniae*: a significant but underrated pathogen in paediatric community-acquired lower respiratory tract infections. Indian J Med Res 2018;147:23–31. 29749357 10.4103/ijmr.IJMR_1582_16 PMC5967212

[2] Kassisse E García H Prada L Prevalence of *Mycoplasma pneumoniae* infection in pediatric patients with acute asthma exacerbation. Arch Argent Pediatr 2018;116:179–85. 29756701 10.5546/aap.2018.eng.179

[3] Dai W Wang H Zhou Q The concordance between upper and lower respiratory microbiota in children with *Mycoplasma pneumoniae* pneumonia. Emerg Microbes Infect 2018;7:92. 29789582 10.1038/s41426-018-0097-y PMC5964150

[4] Cosentini R Tarsia P Canetta C Severe asthma exacerbation: role of acute *Chlamydophila pneumoniae* and *Mycoplasma pneumoniae* infection. Respir Res 2008;9:48. 18513407 10.1186/1465-9921-9-48 PMC2435234

[5] Watanabe H Uruma T Nakamura H The role of *Mycoplasma pneumoniae* infection in the initial onset and exacerbations of asthma. Allergy Asthma Proc 2014;35:204–10. 24801462 10.2500/aap.2014.35.3742

[6] Duenas Meza E Jaramillo CA Correa E Virus and *Mycoplasma pneumoniae* prevalence in a selected pediatric population with acute asthma exacerbation. J Asthma 2016;53:253–60. 26799194 10.3109/02770903.2015.1075548

[7] Shee CD Wheeze and *Mycoplasma pneumoniae*. J R Soc Med 2002;95:132–3. 11872762 10.1258/jrsm.95.3.132 PMC1279480

[8] Esposito S Droghetti R Bosis S Cytokine secretion in children with acute *Mycoplasma pneumoniae* infection and wheeze. Pediatr Pulmonol 2002;34:122–7. 12112778 10.1002/ppul.10139

[9] Defilippi AC Silvestri M Giacchino R Changes in blood eosinophil numbers during *Mycoplasma pneumoniae* infection in wheezing and non-wheezing, atopic and non-atopic children. Pediatr Int 2008;50:718–21. 19261132 10.1111/j.1442-200X.2008.02720.x

[10] Atkinson TP Balish MF Waites KB Epidemiology, clinical manifestations, pathogenesis and laboratory detection of *Mycoplasma pneumoniae* infections. FEMS Microbiol Rev 2008;32:956–73. 18754792 10.1111/j.1574-6976.2008.00129.x

[11] Clyde WA Jr Clinical overview of typical *Mycoplasma pneumoniae* infections. Clin Infect Dis 1993;17 suppl 1:S32–6. 8399935

[12] Wang JY Zheng J Xing HY Determination of Th9 cells and IL-9 in children with *Mycoplasma pneumoniae* infection. Zhongguo Dang Dai Er Ke Za Zhi 2015;17:308–11. 25919545

[13] Shao L Cong Z Li X Changes in levels of IL-9, IL-17, IFN-γ, dendritic cell numbers and TLR expression in peripheral blood in asthmatic children with *Mycoplasma pneumoniae* infection. Int J Clin Exp Pathol 2015;8:5263–72. 26191227 PMC4503099

[14] Chen Z Shao X Dou X Role of the *Mycoplasma pneumoniae*/interleukin-8/neutrophil axis in the pathogenesis of pneumonia. PLoS One 2016;11:e0146377. 26752656 10.1371/journal.pone.0146377 PMC4708980

[15] Yan T Role of anti-inflammatory cytokines in pathogenesis of pediatric *Mycoplasma pneumoniae* pneumonia. J Biol Regul Homeost Agents 2016;30:541–5. 27358146

[16] Wang ZH Li XM Wang YS Changes in the levels of interleukin-17 between atopic and non-atopic children with *Mycoplasma pneumoniae* pneumonia. Inflammation 2016;39:1871–5. 27531365 10.1007/s10753-016-0422-3

[17] Medjo B Atanaskovic-Markovic M Nikolic D Increased serum interleukin-10 but not interleukin-4 level in children with *Mycoplasma pneumoniae* pneumonia. J Trop Pediatr 2017;63:294–300. 28057814 10.1093/tropej/fmw091

[18] Zhao J Zhang W Shen L Association of the ACE, GSTM1, IL-6, NOS3, and CYP1A1 polymorphisms with susceptibility of *Mycoplasma pneumoniae* pneumonia in Chinese children. Medicine (Baltimore) 2017;96:e6642. 28403117 10.1097/MD.0000000000006642 PMC5403114

[19] Moher D Shamseer L Clarke M Preferred reporting items for systematic review and meta-analysis protocols (PRISMA-P) 2015 statement. Syst Rev 2015;4:1. 25554246 10.1186/2046-4053-4-1 PMC4320440

[20] Wells GA Shea B O’Connell D The Newcastle-Ottawa Scale (NOS) for assessing the quality of nonrandomised studies in MetaAnalyses, 2014. Available at: http://www.ohri.ca/programs/clinical_epidemiology/oxford.asp (access date February 1, 2019).

[21] Sutton AJ Duval SJ Tweedie RL Empirical assessment of effect of publication bias on meta-analyses. BMJ 2000;320:1574–7. 10845965 10.1136/bmj.320.7249.1574 PMC27401

